# Complete plastome sequence of *Averrhoa carambola* L. (Oxalidaceae)

**DOI:** 10.1080/23802359.2016.1209095

**Published:** 2016-09-05

**Authors:** Sangjin Jo, Hoe-Won Kim, Young-Kee Kim, Se-Hwan Cheon, Ki-Joong Kim

**Affiliations:** School of Life Sciences, Korea University, Seoul, Korea

**Keywords:** *Averrhoa carambola*, Oxalidaceae, plastome, tropical fruit

## Abstract

In this study, we determined the complete plastome sequence of *Averrhoa carambola* L. (Oxalidaceae) (NCBI acc. no. KX364202). To the best of our knowledge, this is the first reported complete plastome sequence from the order Oxalidales. The gene order and structure of the *A. carambola* plastome are collinear with the typical plastome of land plants. The complete plastome size is 155,965 bp in length and consists of a large single copy region of 87,217 bp and a small single copy region of 17,496 bp, which are separated by a pair of 25,626-bp-long inverted repeat regions. The overall A-T content of the plastome sequence is 61.2%. The plastome contains 111 genes, of which 77 are protein-coding genes, 30 are tRNA genes, and 4 are rRNA genes. Sixteen genes contain one intron and two genes have two introns. A total of 91 simple sequence loci were identified from the genome. Phylogenetic analysis revealed that *A. carambola* is a sister group of *Euonymus japonicus* with 100% bootstrap support.

*Averrhoa carambola* L. is commonly referred to as carambola or starfruit. It is a widely cultivated tropical fruit that originated in tropical Asia (Kim 2011). The fruit of this species is used for both food and medicine. *Averrhoa carambola* belongs to the family Oxalidaceae, which is one of the seven families in the order Oxalidales (Byng et al. 2016). To date, there have been no published plastome sequences for plants in Oxalidales, even though there have been several phylogenetic studies using chloroplast gene markers (Soltis et al. 2000, 2003). Oxalidaceae consists of six genera and approximately 770 species, several of which, such as starfruit (*A. carambola*) and oca (*Oxalis tuberosa*), are plants of economic importance. The complete plastome sequence of *A. carambola* will aid us in developing molecular markers for the identification and improvement of cultivars of this species. In addition, being the first plastome data for the order Oxalidales, the complete plastome sequence of *A. carambola* is expected to become a standard reference for elucidating plastome evolution and phylogenetic relationships in this order.

The leaves of *A. carambola* used in this study were collected from the Korea University greenhouse, where we grew the plants from seeds that were originally collected in Thailand. The plants flowered and fruited in the greenhouse. A voucher specimen was deposited in the Korea University Herbarium (KUS acc. no. 2014-0241). Fresh leaves were ground into powder in liquid nitrogen and the total DNA was extracted using the CTAB method (Doyle & Doyle 1987). The DNAs were further purified by ultracentrifugation and dialysis (Palmer 1986). The genomic DNAs are deposited in the Plant DNA Bank in Korea (PDBK acc. no. 2014-0241). The complete plastome sequence was generated using an Illumina HiSeq 2000 system (Illumina, Inc., San Diego, CA). Annotations were performed using the National Center for Biotechnology Information (NCBI) BLAST, DOGMA (Wyman et al. 2004), and tRNAscan-SE programs (Lowe & Eddy 1997). For the phylogenetic analysis, we selected and downloaded 37 complete plastome sequences based on the APG IV system (Byng et al. 2016) from the NCBI database. All the 37 plastome sequences were from super-rosid plants.

The gene order and structure of the *A. carambola* plastome are similar to those of a typical angiosperm (Shinozaki et al. 1986; Kim & Lee 2004; Yi & Kim 2012). The complete plastome is 155,965 bp in length, and consists of a large single copy (LSC) region of 87,217 bp and a small single copy (SSC) region of 17,496 bp separated by two inverted repeats (IR) of 25,626 bp. The plastome comprises 111 unique genes (77 protein-coding genes, 30 tRNA genes and four rRNA genes). Seven protein-coding, seven tRNA and four rRNA genes are duplicated in the IR regions. The *infA* and *rpl32* genes are pseudogenes. The major portion of the *A. carambola* plastome consists of protein-coding genes (54.8%), tRNA genes (1.8%), and rRNA genes (5.8%). The average A-T content of the plastome is 61.2%, whereas that in the LSC, SSC, and IR regions is 65.7%, 69.8% and 57.5%, respectively. Sixteen genes have one intron and two genes, *ycf3* and *clpP* have two introns. A total of 91 simple sequence repeat (SSR) loci, which can be defined as having more than 10 duplications of simple nucleotide(s), are scattered among the noncoding regions of the genome. Among these, 56, 12 and 23 are mono-SSR, di-SSR, and tri-SSR loci, respectively. Some of these loci will be useful in identifying cultivars of *A. carambola*.

To validate the phylogenetic relationships of *A. carambola* in rosids, we constructed a maximum likelihood (ML) tree by using 38 super-rosid taxa. Phylogenetic analysis was performed on a data set that included 76 protein-coding genes (excluding *infA*, *rpl32*, and *rps16*) and four rRNA genes from the 38 taxa using RAxML v. 7.7.1 (Figure 1; Stamatakis et al. 2008). The 80 gene sequences (82,922 bp in length) were aligned with the MUSCLE program using Geneious v. 6.1.8 (Biomatters Ltd., Auckland, New Zealand). The results showed that *A. carambola* is included in a clade containing plants in the orders Celastrales and Malpighiales (COM clade) with 100% bootstrap support. Similar results were obtained using the APG IV system (Byng et al. 2016). However, the relationships within the COM clade identified in the present study differ from those identified using the APG IV system. A sister-group relationship between Oxalidales and Geraniales was proposed based on the results obtained using the APG IV system. In contrast, a sister-group relationship between Oxalidales and Celastrales is suggested in the present study. Currently, there is only one complete plastome sequence available for each of the orders Oxalidales and Celastrales (Choi & Park 2016). In order to resolve the relationships within the COM clade, further complete plastome sequences are needed from Oxalidales and Celastrales.

**Figure 1.**
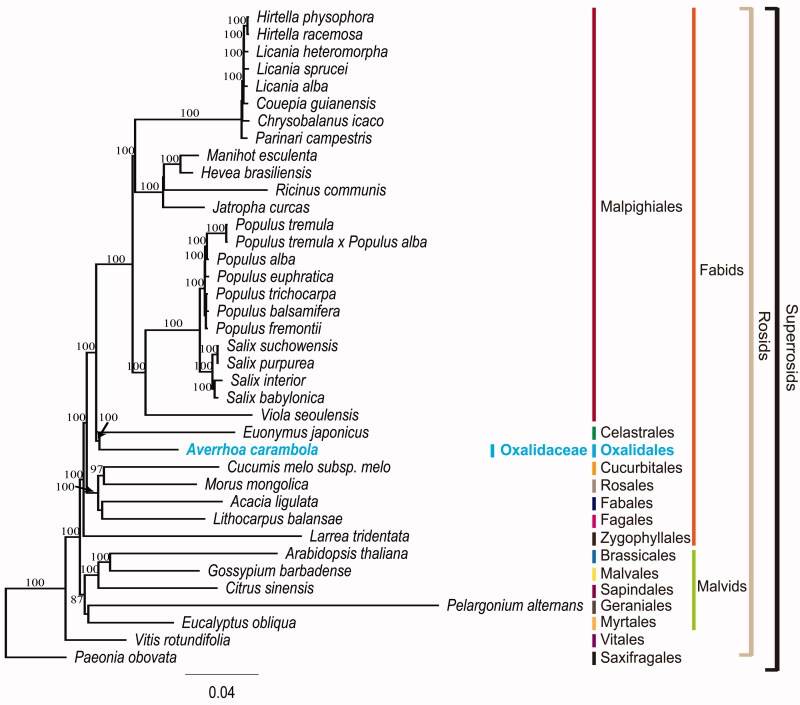
Maximum likelihood (ML) tree based on 76 protein-coding and four rRNA genes from 38 plastid genomes as determined by RAxML. The numbers at each node indicate the ML bootstrap values. Genbank accession numbers of taxa are shown below, *Acacia ligulata* (NC_026134), *Arabidopsis thaliana* (NC_000932), *Averrhoa carambola* (KX364202), *Chrysobalanus icaco* (NC_024061), *Citrus sinensis* (NC_008334), *Couepia guianensis* (NC_024063), *Cucumis melo* subsp. *melo* (NC_015983), *Eucalyptus obliqua* (NC_022378), *Euonymus japonicus* (NC_028067), *Gossypium barbadense* (NC_008641), *Hevea brasiliensis* (NC_015308), *Hirtella physophora* (NC_024066), *Hirtella racemosa* (NC_024060), *Jatropha curcas* (NC_012224), *Larrea tridentata* (NC_028023), *Licania alba* (NC_024064), *Licania heteromorpha* (NC_024062), *Licania sprucei* (NC_024065), *Lithocarpus balansae* (NC_026577), *Manihot esculenta* (NC_010433), *Morus mongolica* (NC_025772), *Paeonia obovata* (NC_026076), *Parinari campestris* (NC_024067), *Pelargonium alternans* (NC_023261), *Populus alba* (NC_008235), *Populus balsamifera* (NC_024735), *Populus euphratica* (NC_024747), *Populus fremontii* (NC_024734), *Populus tremula* (NC_027425), *Populus tremula* × *Populus alba* (NC_028504), *Populus trichocarpa* (NC_009143), *Salix babylonica* (NC_028350), *Salix interior* (NC_024681), *Salix purpurea* (KP019639), *Salix suchowensis* (NC_026462), *Viola seoulensis* (NC_026986) and *Vitis rotundifolia* (NC_023790).
